# Changes in Oesophageal Transit, Macro-Reflux Events, and Gastric Emptying Correlate with Improvements in Gastro-Intestinal Symptoms and Food Tolerance Early Post Sleeve Gastrectomy

**DOI:** 10.1007/s11695-023-06695-z

**Published:** 2023-06-22

**Authors:** Anagi C. Wickremasinghe, Yazmin Johari, Helen Yue, Cheryl Laurie, Kalai Shaw, Julie Playfair, Paul Beech, Geoffrey Hebbard, Kenneth S. Yap, Wendy Brown, Paul Burton

**Affiliations:** 1 Monash University Department of Surgery, Central Clinical School, Monash University, Melbourne, Australia; 2 Oesophago-gastric and Bariatric Unit, Department of General Surgery, The Alfred Hospital, Melbourne, Australia; 3 Department of Nuclear Medicine, The Alfred Hospital, Melbourne, Australia; 4 Department of Gastroenterology, Royal Melbourne Hospital and University of Melbourne, Parkville, VIC 3050 Australia; 5 Department of Medicine, Monash University, Alfred Hospital Campus, Melbourne, VIC 3004 Australia

**Keywords:** Sleeve, Physiology, Bariatric outcome, Clinical trial, Reflux, Gastric emptying, Nuclear scintigraphy, Food tolerance, Gastrointestinal symptoms, Bariatric surgery mechanism

## Abstract

**Purpose:**

There are significant alterations in gastro-intestinal function, food tolerance, and symptoms following sleeve gastrectomy (SG). These substantially change over the first year, but it is unclear what the underlying physiological basis for these changes is. We examined changes in oesophageal transit and gastric emptying and how these correlate with changes in gastro-intestinal symptoms and food tolerance.

**Material and Methods:**

Post-SG patients undertook protocolised nuclear scintigraphy imaging along with a clinical questionnaire at 6 weeks, 6 months, and 12 months.

**Results:**

Thirteen patients were studied: mean age (44.8 ± 8.5 years), 76.9% females, pre-operative BMI (46.9 ± 6.7 kg/m2). Post-operative %TWL was 11.9 ± 5.1% (6 weeks) and 32.2 ± 10.1% (12 months), *p*-value < 0.0001. There was a substantial increase of meal within the proximal stomach; 22.3% (IQR 12%) (6 weeks) vs. 34.2% (IQR 19.7%) (12 months), *p* = 0.038. Hyper-accelerated transit into the small bowel decreased from 6 weeks 49.6% (IQR 10.8%) to 42.7% (IQR 20.5%) 12 months, *p* = 0.022. Gastric emptying half-time increased from 6 weeks 19 (IQR 8.5) to 12 months 27 (IQR 11.5) min, *p* = 0.027. The incidence of deglutitive reflux of semi-solids decreased over time; 46.2% (6 weeks) vs. 18.2% (12 months), *p*-value < 0.0001. Reflux score of 10.6 ± 7.6 at 6 weeks vs. 3.5 ± 4.4 at 12 months, (*p* = 0.049) and regurgitation score of 9.9 ± 3.3 at 6 weeks vs. 6.5 ± 1.7, *p* = 0.021 significantly reduced.

**Conclusions:**

These data demonstrate that there is an increase in the capacity of the proximal gastric sleeve to accommodate substrate over the first year. Gastric emptying remains rapid but reduce over time, correlating with improved food tolerance and reduced reflux symptoms. This is likely the physiological basis for the changes in symptoms and food tolerance observed early post-SG.

**Graphical Abstract:**

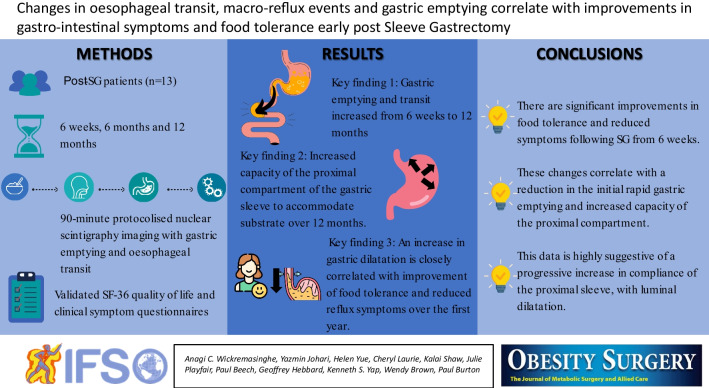

## Introduction

Sleeve gastrectomy (SG) has rapidly gained popularity due to its ability to induce and sustain substantial weight loss; however, the underlying physiological effects of the procedure have not been adequately delineated. In particular, early in the post-operative period there are significant alterations to gastro-intestinal symptoms, sensations and food intake [1]. These appear to improve over time to the 12-month mark with increasing food tolerance [2]. It is unclear which physiological mechanisms mediate these and how they change over time. Understanding the basis of evolution in sensations would be of significant advantage to clinicians following-up and counselling these patients.

Following SG, substantial physiological changes have been observed and a new paradigm of oesophageal and gastric function has been developed [3]. The weight loss mechanisms are multifactorial and incompletely defined, but appear to be associated with both hormonal alterations and changes in gastric emptying [4]. Accelerated gastric emptying after SG and has been reported by several groups [5–8]. This rapid emptying is driven by an oesophageal-mediated mechanism of isobaric pressurisations in the vertical compartment of the sleeve with repeat peristalsis [3]. It appears that the mechanisms of weight loss are associated with rapid gastric transit. Furthermore, previous studies showed that longer gastric emptying half-times are associated with poor weight loss [9].

Prior literature has evaluated gastric emptying changes before and after SG surgery and have not focussed on changes in gastric emptying in the first year after surgery [10]. A significant consideration is that there is progressive dilatation of the sleeve over time, enabling patients to ingest larger volumes of food more frequently; this is supported by long-term data by Himpens et al. (2010), who have demonstrated dilatation of the sleeve and significant alterations following SG [11]. However, further studies are warranted to evaluate these changes. It would be of significant benefit to understand the physiological changes and how these evolve and correlate with patient-reported symptoms and outcomes.

We hypothesised that alterations and increases in reflux symptoms would be noted early in the post-operative period at around 6 weeks, and these would have improved by 6 and 12 months with gastric emptying remaining static. Therefore, we aimed to determine whether from 6 weeks to 1 year there were alterations in oesophageal transit, gastro-oesophageal reflux, and gastric emptying. Additionally, we wished to examine whether alteration in symptoms correlated with changes in physiology. A secondary aim was to evaluate changes out to 3 years in the same physiological and clinical parameters.

## Methods

Ethics approval for this study was obtained from the Alfred Human Research and Ethics Committee (HREC) no. 380/16. All participants gave informed written consent.

### Participants

Thirteen participants undergoing laparoscopic sleeve gastrectomy for morbid obesity were enrolled in this study and were followed up at 6 weeks, 6 months, and 12 months post-surgery. Recruitment of participants took place from a single centre in Melbourne. Inclusion criteria included those aged 18–65 years. Exclusion criteria were those who had previous bariatric or gastric surgery other than the sleeve gastrectomy, substantial adverse symptoms requiring further investigation, pregnancy or breast feeding and use of medication to modify gastric motility or known abnormality of gastric motility.

### Surgical Technique

Sleeve gastrectomy was performed by one surgeon and the technique has been previously described [12]. Surgery involved standardised dissection and mobilisation of the short gastric vessels. The stomach was fully mobilised from the pylorus to the angle of His using tri-staplers (ECHELON FLEX™ GST system), commenced 4 cm from the pylorus over a 36-French bougie. Follow-up was along standard lines for sleeve gastrectomy with post-operative follow-up including scheduled visits, and detailed written information was provided concerning eating approach and textures of food able to be consumed.

### Patient Reported Outcomes

All patients completed a validated standardised questionnaire previously described [13].

### Nuclear Medicine Scintigraphy Studies

An oesophageal transit and gastric emptying study was performed on patients following an overnight fast. All patients undergoing nuclear scintigraphy had their procedures performed in the same room and at the same temperature. Nuclear scintigraphy was performed using a Siemens Symbia™ Evo Excel Gamma Camera.

#### Meal

Patients were required to consume a radio-labelled semi-solid porridge meal consisting of 30g of instant porridge, 100 mL full cream milk microwaved, which was mixed with 40MBq of Tc-99m calcium phytate and one teaspoon of sugar.

#### Oesophageal Transit and Gastric Emptying Study

The first part of the study was to assess semi-solid oesophageal transit with the patient standing. Two semi-solid swallows were conducted each containing three-quarter tablespoon of radio-labelled porridge. Patients were requested to swallow in one attempt without provoking another swallow. Dynamic images were taken 1 s per frame for 60 s from the posterior projection.

Following this, the second part of the study assessed gastric emptying, after patients consumed the remaining meal over 5 min. Patients were imaged in the supine position in the left anterior oblique 30° projection, the images were taken 5 s per frame for 90 min.

The third part of the study assessed liquid oesophageal transit swallows. Two liquid swallows containing 10MBq of Tc-99m Calcium Phytate in 10 ml of water were administered orally by a syringe in the supine position on a radiolucent imaging table. Images were taken every second for 60 s in the posterior projection.

#### Image Processing and Analysis

For both gastric emptying and oesophageal transit studies, the radioactive counts were drawn around the oesophagus, neo-stomach (i.e. including proximal and distal stomach) and small bowel; these were defined as the regions of interest (ROI). Normal oesophageal transit was defined as the complete clearance across the esophagogastric junction by progressive antegrade transit without reflux in the 1 min. Delayed transit was defined as any noticeable hold-up of the bolus or evidence of reflux back into the oesophagus. After the 90 min, any residual activity retained in the oesophagus and stomach were quantified using the first 2 min acquisition frame compared to the residual activity in the final 2 min period. Radioactive counts were represented as a function of time in a time-activity curve (TAC) over 60 s (one image per second). The sleeve shapes were classified into three different patterns of intragastric meal distribution: proximal (dilated portion of the proximal sleeve), antral (dilated portion of the antrum), and uniform (tubular-shaped sleeve). All images were processed on a General Electric Xeleris Functional Imaging Workstation.

### Statistical Analysis and Data Management

Data collected from the participants were compiled and entered into a database designed for the purpose of this study using Microsoft Access (Microsoft Corp, Redmond, WA, USA). All statistical analysis was performed using GraphPad Prism v.9 (GraphPad Software Inc, USA). For the analysis of categorial data, chi-square test was used. For the assessment across multiple groups, where appropriate a one- way ANOVA was used, followed by a Tukey’s post hoc test to determine the difference between the group means. Additionally, a Pearson’s correlation was conducted to quantify the direction and strength between post-operative weight loss and gastric emptying measures. The significance of *p*-value was set at < 0.05.

## Results

### Patient Demographics

A total of 13 trial patients had a post-operative scan at 6-week, 6-month, and 12-month follow-up; details are shown in Table 1. Additionally, 11 obese controls were included for illustrative purposes. The trial patients had a mean baseline age of 44.8 ± 8.5 years, with the majority of trial patients being female (76.9%). Weight loss was significant at the 12-month follow-up (%TWL 6 weeks 11.9 ± 5.1% vs. 12 months 32.2 ± 10.1%, *p*-value < 0.0001).

**Table 1:** Patient demographics

	Obese controls (*n* = 11)	Baseline	6 weeks	6 months	12 months	*p*-value*	*p*-value^^^	*p*-value^¶^
Age (years)	40.7 ± 13.0	44.8 ± 8.5						
Male/female	1/10	3/10						
Start weight (Kg)	121.0 ± 16.3	133.1 ± 22.9						
Start BMI (Kg/m^2^)	45.8 ± 6.6	46.9 ± 6.7						
Diabetes, *n* (%)	-	0						
Hypertension, *n* (%)	-	5 (38.5)						
Obstructive sleep apnea, *n* (%)	-	3 (23.1)						
Reflux, *n* (%)	-	4 (30.8)						
PPI use, (%)	-	3 23.1)						
*Post-op*								
Weight loss (Kg)			16.4 ± 9.8	33.6 ± 13.3	42.5 ± 17.5	**0.017**	0.872	**0.001**
Weight at follow-up (Kg)			116.7 ± 16.9	98.8 ± 15.7	87.4 ± 17.6	0.090	0.435	**0.090**
BMI at follow-up (Kg/m^2^)			41.1 ± 4.8	34.4 ± 4.0	30.8 ± 4.2	**0.016**	0.342	**< 0.0001**
Percent excess weight loss			25.5 ± 9.5	55.4 ± 17.6	72.6 ± 23.9	**0.007**	0.470	**< 0.0001**
Percent total body weight loss			11.9 ± 5.1	24.9 ± 7.2	32.2 ± 10.1	**0.015**	0.265	**< 0.0001**

Values expressed as mean ± SD, *p-*values for comparison amongst the groups based on the different time points using one-way ANOVA

The values that have been bolded indicate significant *p*-values

*p-value between 6 weeks vs. 6 months

^*p*-value between 6 months vs. 12 months

^¶^*p*-value between 6 weeks vs. 12 months

### Intragastric Meal Distribution

**Fig. 1:**
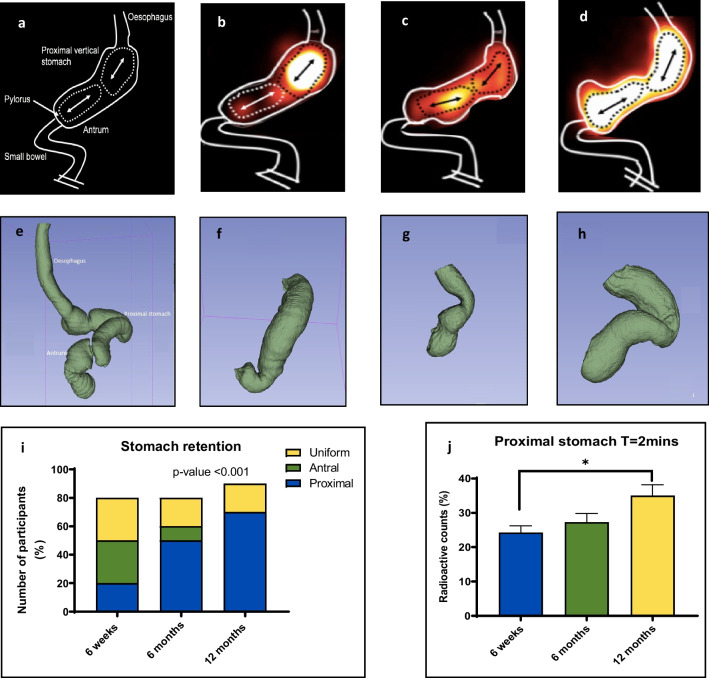
Intragastric meal distributions in the study cohort patients observed over time. **a**–**d** are illustrative drawings demonstrating nuclear scintigraphy images with presumed anatomy and regions of interest (ROI) as drawn. **b** proximal IMD **c** antral IMD and **d** uniform IMD. Panels **e** to **h** illustrate 3D reconstruction volumetric computed tomography of the gastric sleeve. **f** represents proximal dilated stomach **g** represents antral dilated stomach **h** represents uniformly dilated stomach

At the start of the scan (T = 2 min), three different patterns of intragastric meal distribution (IMD) were observed: proximal, antral, and uniform retention (Fig. 1a–d). At 6 weeks post-surgery, the majority of patients (64%, *n* = 8) were classified as having a uniformly distributed meal. After 12 months 70% (*n* = 9) of patients had developed a dilation of the proximal compared to 6 weeks (*p*-value < 0.0001). To objectively confirm this finding of proximal meal retention, we quantified the radioactive counts within the different segments of the stomach. Figure 1j shows there was a significant increase of meal retention within the proximal stomach from 22.95% (IQR 12.02%) at 6 weeks to 34.19% (IQR 19.73%) at 12 months (*p*-value 0.038).

### Oesophageal Bolus Clearance and Deglutitive Reflux

**Fig. 2:**
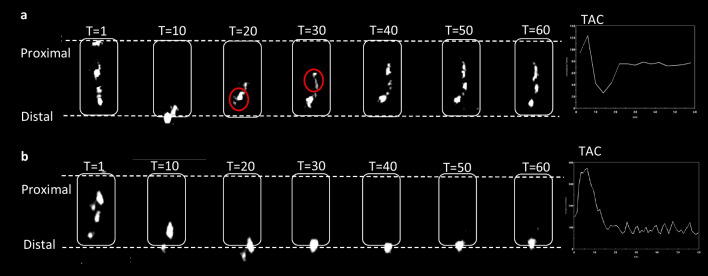
Schematic of triggered deglutitive reflux on a 60 s nuclear scintigraphy oesophageal swallow study. Upper dotted line = manubrium, lower dotted line = xiphisternum. **a** Bolus-induced deglutitive reflux, majority of the radioactive food bolus moved to the mid oesophagus. The red circle represents the start of reflux. **b** No reflux, majority of the food bolus was seen moving from the proximal oesophagus to the distal oesophagus within 60 s after ingestion

On nuclear scintigraphy observation, patients showed deglutitive reflux and delayed oesophageal transit during liquid and semi-solid swallows (Fig. 2). At 6 weeks post-surgery, the majority of patients 46.2% (*n* = 6) experienced triggered deglutitive reflux on semi-solid swallows. Over-time, this phenomenon significantly decreased, with only 18.2% (*n* = 2) of patients demonstrating reflux at 12 months (*p*-value < 0.0001). There were no significant changes in delayed swallows for bolus transit.

### Gastric Emptying

Over time, the rapid gastric emptying half-time slowed appreciably, although not to a normal level. Gastric emptying half-time were as follows: 19.00 (IQR 8.50) min (6 weeks) vs. 27.00 (IQR 10.50) min (6 months) vs. 27.00 (IQR 11.50) min (12 months), *p*-value 0.027.

### Gastric Clearance and Small Bowel Delivery

**Fig. 3:**
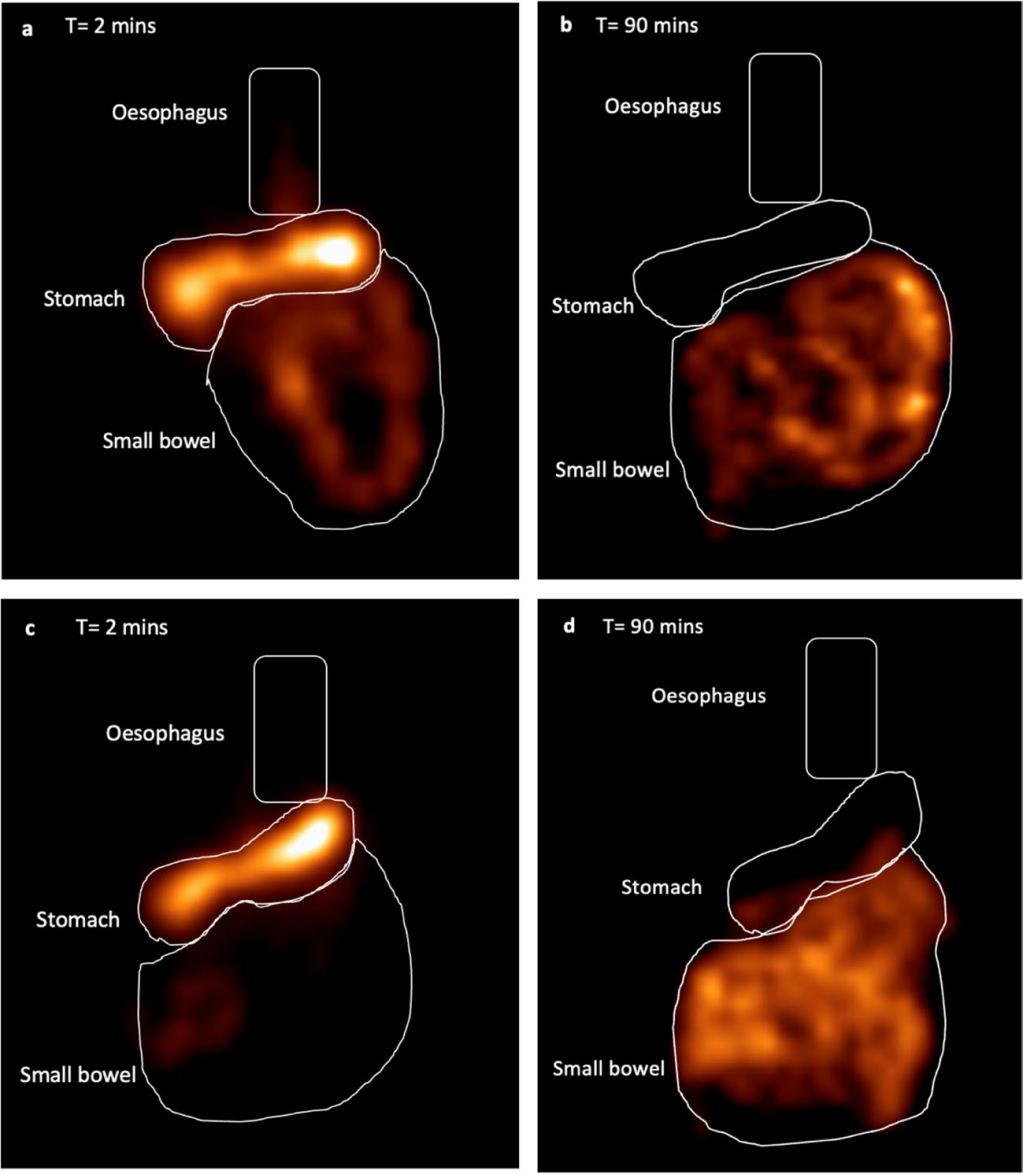
Nuclear Scintigraphy gastric clearance and intestinal delivery. **a**–**d** are **s**chematic representations of proportional emptying of a patient at 6 weeks (a and b) and 12 months (c and d) post sleeve gastrectomy. **a** illustrates a uniform retained stomach with hyper-acceleration of emptying into the small bowel at T = 2 min (6 week scan). **b** illustrates most (97.2%) of the meal in the small bowel at T = 90 min (6 week scan). **c** illustrates proximal retention and little emptying into the small bowel at T = 2 min (12 month scan). **d** illustrates an increase in meal retention in the stomach and lesser (91.1%) emptying into the small bowel T = 90 min (12 month scan)

On the initial acquisition frame (T = 2 min), hyper-acceleration of radioactive meal into the small bowel significantly reduced over time 6 weeks 49.59% (IQR 10.81%) vs. 12 months 42.67% (IQR 20.47%), *p*-value 0.022). The proportion of meal retained in the overall stomach at the conclusion of the 90 min study increased over time; however, this was not of significance (Table 2). Figure 3 illustrates each of the esophago-gastric compartments emptying at 6 weeks and 12 months.

**Table 2:** Physiology data using nuclear scintigraphy

Variable	Obese controls	6 weeks	6 months	12 months	*p*-value^$^	*p*-value*	*p*-value^^^	*p*-value^¶^
Oesophageal transit study								
Delay in transit of liquid, *n* (%)	9 (81.8)	3 (27.3)	2 (18.2)	4 (30.8)	**< 0.0001**	> 0.999	0.878	> 0.999
Delay in transit of semi-solids, *n* (%)	5 (45.5)	2 (18.2)	2 (18.2)	3 (27.3)	**< 0.0001**	0.878	0.878	> 0.999
Deglutitive reflux of liquids, *n* (%)	2 (18.2)	8 (61.5)	6 (46.2)	7 (53.8)	**< 0.0001**	0.987	> 0.999	> 0.999
Deglutitive reflux of semi-solids, *n* (%)	10 (90.9)	6 (46.2)	4 (30.8)	2 (18.2)	**< 0.0001**	> 0.999	> 0.999	**< 0.0001**
Gastric transit study								
Gastric emptying half-time, median (IQR) minutes	75.80 (45.19)	19.00 (8.50)	27.00 (10.50)	27.00 (11.50)	**0.012**	0.116	> 0.999	**0.027**
Proximal sleeve emptying half-time, median (IQR) minutes	-	20.00 (11.25)	24.00 (13.00)	29.00 (10.00)	-	0.486	> 0.999	0.127
Antral sleeve emptying half-time, median (IQR) minutes	-	19.00 (17.00)	26.00 (13.00)	22.00 (19.50)	-	> 0.999	> 0.999	0.989
*Proportion of counts at T = 2 min,* median *(IQR)*								
Oesophagus (%)	5.37 (4.32)	5.10 (6.12)	4.29 (5.94)	3.47 (5.03)	0.675	> 0.999	> 0.999	0.466
Overall stomach (%)	74.30 (18.50)	42.68 (9.94)	42.69 (12.23)	52.55 (20.08)	**0.008**	0.955	0.686	**0.006**
Proximal (%)	-	22.95 (12.02)	26.98 (12.80)	34.19 (19.73)	**-**	> 0.999	0.221	**0.038**
Antral (%)	-	20.21 (9.42)	21.00 (6.17)	20.85 (7.68)	**-**	> 0.999	> 0.999	> 0.999
Small bowel (%)	18.66 (13.42)	49.59 (10.81)	50.08 (13.33)	42.67 (20.47)	**0.006**	> 0.999	0.791	**0.022**
*Proportion of counts at T = 90 min, median (IQR)*								
Oesophagus (%)	2.20 (1.60)	0.53 (0.41)	0.48 (0.35)	0.56 (0.61)	**0.045**	> 0.999	> 0.999	> 0.999
Overall stomach (%)	32.70 (24.60)	2.70 (4.07)	3.72 (3.20)	5.77 (6.84)	**0.002**	> 0.999	0.217	0.083
Proximal (%)	-	1.60 (2.39)	2.57 (1.91)	4.60 (4.25)	**-**	0.780	0.120	**0.004**
Antral (%)	-	0.96 (2.54)	1.94 (2.01)	2.96 (5.31)	**-**	> 0.999	0.397	0.054
Small bowel (%)	65.10 (25.90)	96.60 (4.49)	95.70 (3.77)	94.08 (7.19)	**0.018**	> 0.999	0.296	0.080

Values expressed as median and inter quartile range (IQR), *p-*values for comparison amongst the groups based on the different time points using one-way ANOVA

The values that have been bolded indicate significant *p*-values

^$^overall *p*-value between obese controls and post-op sleeve time points

*p-value between 6 weeks vs. 6 months

^*p*-value between 6 months vs. 12 months

^¶^*p*-value between 6 weeks vs. 12 months

### Effects of Post-Operative Weight Loss on Gastric Emptying Measures

Several anthropometric variables were significantly associated with gastric emptying measures post-sleeve gastrectomy (Table 3). Post-operative weight (Kg) was the only anthropometric measure to be positively associated with gastric emptying half-time (minutes) (r = 0.7, *p*-value 0.015). Additionally, post-operative weight was also associated with overall stomach (r = 0.3, *p*-value 0.042) and proximal stomach (r = 0.3, *p*-value 0.053) proportion at T = 2 min of the scan. Furthermore, post-operative BMI (Kg/m^2^) was observed to be positively associated with overall stomach (r = 0.4, *p*-value 0.002), proximal stomach (r = 0.3, *p*-value 0.024) and negatively associated with small bowel (r = −0.4, *p*-value 0.012) proportion at T = 2 min of the scan. The proportion of proximal stomach counts at T = 90 min of the scan was also significantly associated with post-operative BMI.

**Table 3:** Anthropometrics correlation with gastric emptying measures

Variable	Post-op Weight		Post-op BMI		%TWL		%EWL		Post-op time duration (months)	
	r	*p*-value	r	*p*-value	r	*p*-value	r	*p*-value	r	*p*-value
Gastric emptying half-time	0.7	**0.015**	0.6	0.048	−0.1	0.440	−0.02	0.878	0.2	0.133
*Proportion of counts at T = 2 min*										
Overall stomach	0.3	**0.042**	0.4	**0.002**	−0.3	**0.001**	−0.1	0.527	0.4	0.010
Proximal stomach	0.3	**0.053**	0.3	**0.024**	0.2	0.220	−0.1	0.474	0.3	**0.045**
Antral	−0.03	0.849	−0.2	0.123	0.05	0.744	−0.1	0.692	0.1	0.700
Small Bowel	0.3	0.112	−0.4	**0.012**	−0.3	**0.021**	0.1	0.499	−0.3	0.118
*Proportion of counts at T = 90 min*										
Overall stomach	0.1	0.561	−0.2	0.126	0.2	0.105	−0.1	0.787	0.4	**0.013**
Proximal stomach	0.1	0.693	0.4	**0.019**	0.3	0.095	−0.1	0.549	0.4	**0.012**
Antral	0.03	0.853	−0.2	0.284	0.1	0.302	−0.1	0.735	0.3	**0.024**
Small bowel	0.1	0.651	0.2	0.255	−0.2	0.157	0.1	0.582	−0.4	**0.013**

The values that have been bolded indicate significant *p*-values

%TWL was negatively associated with proportion of the overall stomach (r = −0.3, *p*-value 0.001) and small bowel (r = −0.3, *p*-value 0.021) at T = 2 min. Proportion of proximal stomach retention at the T = 2 min was positively associated with post-operative time (r = 0.3, *p*-value 0.045). Additionally, post-operative time was positively associated with proportion of counts in the overall stomach, proximal stomach and antrum and negatively associated with the small bowel. However, there were no associations found between %EWL and gastric emptying measures.

### Patient-Reported Outcomes

#### Symptoms

##### Reflux

The overall reflux scores were maintained at a low level, with a significant decrease over the follow-up period from 6 weeks to 6 months to 12 months (10.6 ± 7.6 vs. 6.7 ± 7.7 vs. 3.5 ± 4.4, *p*-value 0.049), with 0 representing no reflux and 72 representing maximum severity. Twenty-nine percent of patients at 6 months compared to 10% of patients at 12 months reported experiencing heart burn on most days (*p*-value 0.046). The use of anti-reflux medication did not significantly change between 6 weeks and 12 months (36% vs. 27%, *p*-value 0.734).

##### Regurgitation and Vomiting

There was a substantial decrease in the frequency of regurgitation experienced by patients between 6 weeks and 12 months (44% vs. 20%, *p*-value < 0.0001). Additionally, the composite regurgitation scores significantly decreased from 6 weeks to 12 months (9.9 ± 3.3 vs. 6.5 ± 1.7, *p*-value 0.021), with 0 representing no regurgitation and 45 representing maximum regurgitation.

##### Dysphagia

The mean dysphagia scores were low and did not change significantly (8.1 ± 6.4, 9.2 ± 9.4, ± 9.4, 5 ± 5.5, *p*-value 0.4216), with 0 representing no dysphagia to any food and 45 being total dysphagia, unable to swallow water. At 12 months, 80% of patients never experienced dysphagia.

##### Abdominal Bloating

**Fig. 4:**
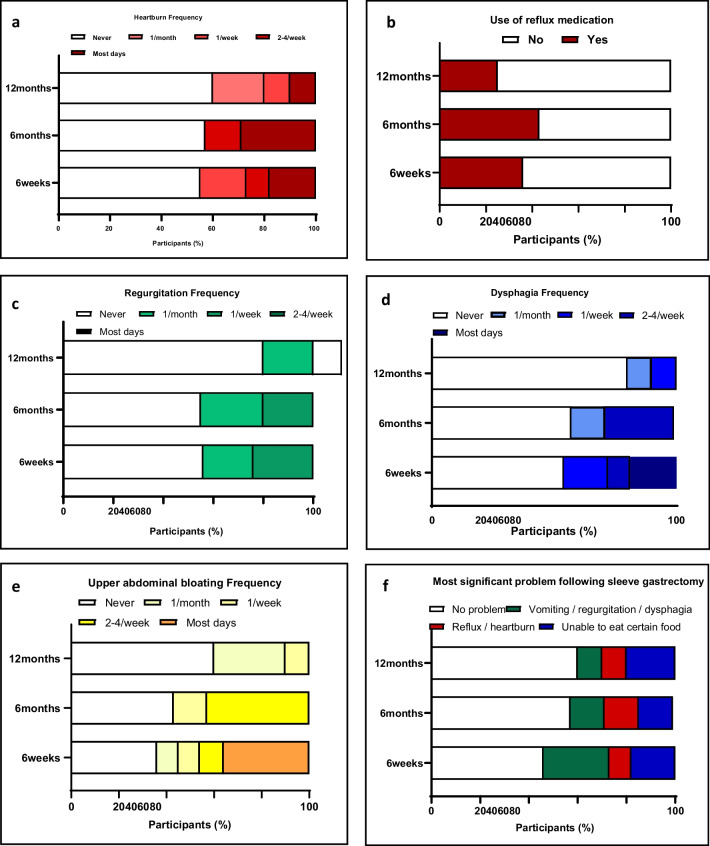
Patient reported outcome measures on adverse symptoms. Responses to **a** ‘heartburn frequency’, **b** ‘use of reflux medication’, **c** ‘regurgitation frequency’, **d** ‘dysphagia frequency’, **e** ‘upper abdominal bloating’, and **f** ‘most significant problem following sleeve gastrectomy’

The frequency of abdominal bloating was variable, with majority of the patients (36%) reporting abdominal bloating most days at 6 weeks (Fig. 4e). There was a significant increase in those never experiencing bloating symptoms between the 6 weeks and 12 months (36% vs. 60%, *p*-value 0.034).

##### Most Troublesome Symptom

The most troublesome symptom or complaint was vomiting/regurgitation, with a significant difference between 6 weeks and 12 months (27% vs. 9%, *p*-value < 0.0001). There were no major issues for 46% of patients at 6 weeks, which significantly increased to 60% of patients at 12 months (*p*-value < 0.0001).

### Food Tolerance

**Fig. 5:**
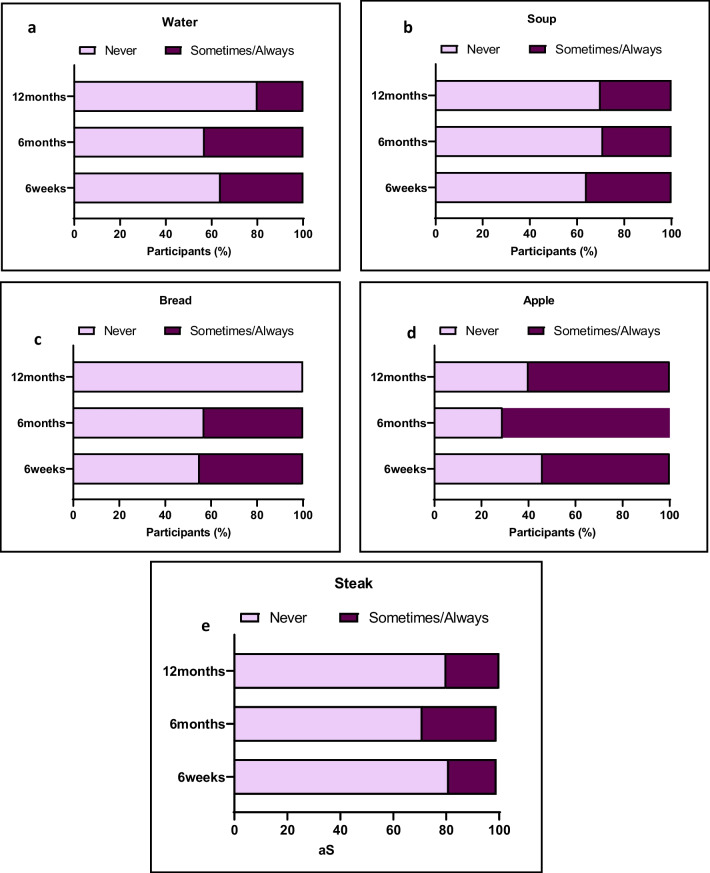
Patient-reported outcome measures on food tolerance. Patients’ ability to consume foods of different texture. For each food type listed, patients were asked if when they consumed these foods they ‘never regurgitated’, ‘sometimes regurgitated/always regurgitated’. Responses to **a** “Water”, **b** “Soup”, **c** “Bread”, **d** “Apple”, **e** “Steak”

Patients were able to tolerate most foods listed in Fig. 5. Post-surgery most patients at each of the follow-ups always had trouble consuming liquids such as water. Soft textured foods were tolerated well at all time points following surgery. Fifty-seven percent of patients always had trouble consuming thicker-textured food such as steak at 6 months; however, this declined to 20%, where patients sometimes had trouble consuming steak (*p*-value < 0.0001).

### Satiety

The mean satiety scores at each of the mealtimes are shown in Table 4. On average the patients reported to be ‘satisfied’ prior to each meal at the 6 weeks’ time point. However, this significantly changed from 6 weeks to 12 months where patients were a ‘little bit hungry’ at lunch (6.1 ± 2 vs. 4.2 ± 1.5 *p*-value 0.025) and dinner (6.8 ± 1.8 vs. 4.5 ± vs. 1.9 *p*-value 0.018).

**Table 4:** Patient reported outcome measures

	6 weeks	6 months	12 months	*p*-value*	*p*-value^^^	*p*-value^¶^
Symptom scores						
Reflux score	10.6 ± 7.6	6.7 ± 7.7	3.5 ± 4.4	0.429	0.429	**0.049**
Regurgitation score	9.9 ± 3.3	7.4 ± 2.9	6.5 ± 1.7	0.161	0.768	**0.021**
Dysphagia score	8.1 ± 6.4	9.2 ± 9.4	5 ± 5.5	**0.945**	0.447	0.564
Hunger scores						
Breakfast score	5.1 ± 2.9	3.7 ± 1.9	4.2 ± 1.6	0.341	0.871	0.609
Lunch score	6.1 ± 2	4.1 ± 1.2	4.2 ± 1.5	**0.022**	0.992	**0.025**
Dinner score	6.8 ± 1.8	4.1 ± 1.6	4.5 ± 1.9	**0.005**	0.833	**0.018**

Values expressed as median and inter quartile range (IQR), *p-*values for comparison amongst the groups based on the different time points using one-way ANOVA

The values that have been bolded indicate significant *p*-values

*p-value between 6 weeks vs. 6 months

^*p*-value between 6 months vs. 12 months

^¶^*p*-value between 6 weeks vs. 12 months

### Satisfaction

Over all patients were highly satisfied with the LSG procedure and did not change over the follow-up time points. The mean satisfaction score was as follows: 9.8 ± 0.6 vs. 9.6 ± 0.7 vs. 9.8 ± 0.6, *p*-value 0.897; this score was out of 10. When asked if they would undergo surgery again, patients were highly likely to undergo the surgery, and this did not change over time. Overall patients were willing to say they would *definitely* undergo surgery again (97% vs. 92% vs. 98.2%, *p*-value 0.986).

## Discussion

We have conducted a prospective observational study with repeated measures of gastric physiology and upper gastrointestinal symptoms and found (1) an increase in the capacity of the proximal compartment of the gastric sleeve to accommodate food over time, (2) gastric emptying and hyper-accelerated transit remain rapid but fell over time, and lastly (3) these physiological changes closely correlate with improved food tolerance and reduced reflux symptoms over the first year.

We observed three patterns of intragastric meal distribution following SG. Initially, meal distribution was even between the proximal and distal stomach; however, over time, patients developed a pattern of proximal gastric dilation and meal retention. This is most likely due to an increase in the compliance of the sleeve. That would seem most attributable to the resolution of post-operative oedema, although the known episodes of isobaric pressurisation in the sleeve may also contribute to dilatation of the region and a reduction in reflux events.

The rapid gastric emptying significantly slowed from 6 weeks to 12 months, but remained rapid in comparison to anatomically normal stomachs (40–70 min) [14]. Our findings of gastric emptying half-time are in concordance with our previous research, demonstrating rapid gastric emptying is a biomarker for successful weight loss following sleeve gastrectomy [9]. Rapid gastric emptying is likely a mechanism associated with the induction of satiety and weight loss as less rapid emptying is associated with reduced weight loss [15–17].

We observed several positive associations of weight loss parameters with gastric emptying measures. An increase in post-operative weight was associated with an increase in the gastric emptying half-time. A longer duration from post-operative time was also associated with an increase in the proportion of meal retained in the proximal stomach. Lastly, we also found lower %TWL, was associated with an increased proportion of meal retained in the overall stomach.

Vomiting/regurgitation was the most troublesome symptom or problem reported by 28% of patients at the 6 week time point. However, this problem improved over time with fewer patients experiencing this at 6 months (14%) and 12 months (9%). Like our findings, a study by Coluzzi et al. (2016), demonstrated a similar proportion of patients (10%) experiencing regurgitation with 65% of patients never experiencing it at 1 year [2]. Whereas a study done by Kvehaugen A and Farup. Per G (2018) showed over 60% of their patient cohort experienced vomiting/regurgitation at least twice per week, which was vastly different to our patients (42%) [18]. Our study separately assessed for post-prandial vomiting/regurgitation and reflux relating to acidic sensations; whereas their study combined vomiting/regurgitation/reflux, which may explain the substantial differences in frequency of symptoms observed between the two studies.

Within the first 6 months post-gastric sleeve, the intraluminal pressures in the sleeve may overcome the resting pressure of the lower oesophageal sphincter that may lead to post-prandial regurgitation/vomiting [19]. Furthermore, with the increase in gastric compliance over time, this may likely improve this symptom. Therefore, these data indicate that vomiting/regurgitation can be minimised by eating appropriate food in small volumes [20]. This also helps set expectations for patients pre-operatively.

Overall, the mean reflux scores were less than 15 out of 72 at all three-time points following SG. The highest reflux was 10.6 out of 72 at the 6-week time point which significantly decreased by 12 months. Deglutitive reflux of semi-solids on nuclear scintigraphy also significantly decreased from 6 weeks to 12 months. This could be due to the significant weight loss with decreased visceral adiposity, thereby decreasing the intragastric pressure over time and the dilation of the proximal stomach increasing compliance of the region [21].

Similarly to our findings of intragastric meal distribution, Lazoura et al. (2011) suggested three different radiological patterns of the gastric sleeve [22]. However, it was a brief radiological anatomic study using a single liquid contrast performed day one, unlike our study which used dynamic functional images with repeated measures. Vigneshwaran et.al (2016) conducted a prospective observational study looking at the changes in gastric emptying following SG [23]. Their post-operative time points were 3 to 6 months and 12 months. Their study used rice dumplings instead of porridge and no details of the technique image acquisition were included. The emptying half-time at the 3- to 6-month mark was similar to that of our findings; however, their emptying half-times did not change appreciably over time. This is likely due to the initial measurement time point being relatively late (3 months), when most of the physiological adaption may have occurred.

The strengths of this study centre around its combination of a prospectively monitored with matched sleeve gastrectomy patients. We have conducted a more detailed analysis of the nuclear scintigraphy scans, using tailored algorithms, with additional physiological measures beyond the simple measurement of gastric emptying half-time. Our study used an earlier time point (6 weeks) than other studies, and this has allowed us to identify significant physiological changes that occur in the early post-operative period. Additionally, we were able to demonstrate an increase in dilatation occurring in the proximal sleeve by performing a detailed analysis. Validated questionnaires were used to correlate symptom changes and patient-reported measures with emptying and transit.

Future directions will evaluate whether early symptoms and food tolerance can be improved through strategies specifically designed to target the observed physiological changes. In situations where abnormal progress is noted, nuclear scintigraphy can be applied as a diagnostic test to understand whether sleeve function is as expected. An additional important area is to attain measures of changes over longer periods of time to better understand the drivers of luminal dilatation. This would link to evaluations of strategies aiming to improve longer-term outcomes, such as a focus on good eating behaviours aiming to avoid over pressurisation of the proximal gastric sleeve.

## Conclusions

This prospective study has demonstrated significant improvements in food tolerance and reduced symptoms following SG from 6 weeks. These changes correlate with a reduction in the initial rapid gastric emptying and increased capacity of the proximal compartment. This data is highly suggestive of a progressive increase in compliance of the proximal sleeve, with luminal dilatation. Clinicians can use these data to inform their management of SG patients as it provides a physiological framework to correlate with patients’ symptoms and sensations.
